# Pharmacists as immunizers in Lebanon: a national survey of community pharmacists’ willingness and readiness to administer adult immunization

**DOI:** 10.1186/s12960-021-00673-1

**Published:** 2021-10-24

**Authors:** Dalal Youssef, Linda Abou-Abbas, Suzan Farhat, Hamad Hassan

**Affiliations:** 1 Preventive Medicine Department, Ministry of Public Health, Beirut, Lebanon; 2 Research Center for Population Health (BPH), Institut de santé publique d’épidémiologie et de développement (ISPED), Bordeaux University, Bordeaux, France; 3 Neuroscience Research Center, Faculty of Medical Sciences, Lebanese University, Beirut, Lebanon; 4 Epidemiological Surveillance Unit, Ministry of Public Health, Beirut, Lebanon; 5 Lebanese University, Beirut, Lebanon; 6 Ministry of Public Health, Beirut, Lebanon

**Keywords:** Community pharmacists, Immunization, Willingness, Readiness, Lebanon

## Abstract

**Background:**

Since the focus of healthcare has shifted toward prevention, pharmacists were highly encouraged to expand their practice to include immunization services. Our study aimed to assess the knowledge, attitudes and beliefs of community-based Lebanese pharmacists, in addition to their willingness to expand their practice scope to include vaccine administration.

**Methods:**

A cross-sectional study was conducted during the phase preceding the arrival of the COVID-19 vaccine in Lebanon between 1 and 31st December 2020. Using a stratified random sampling method, data were collected from Lebanese community pharmacists (CPs) through an online survey that included information on socio-demographic characteristics, clinical experience, willingness to administer vaccines, knowledge about vaccination, attitudes towards immunization, reasons supporting utilizing pharmacists as immunizers and the requested elements to incorporate immunization in pharmacists’ practice scope. Multivariable analyses were performed to identify the factors associated with knowledge.

**Results:**

A total of 412 community pharmacists participated in this survey. Of the total, 66.5% of the surveyed CPs are willing to administer vaccines. The majority of them (89.8%) had an overall good level. Out of all, 92.7% showed a positive overall attitude score toward immunization, 95.4% agreed that community pharmacists can play an important role in advertising and promoting vaccination. The main needed elements for implementing immunization services in pharmacies listed by participants were: support of health authorities (99.3%), statutory allowance (82.8%), patient demand (95.4%), pharmacist’s interest (96.1%) and continuous education and training workshops on immunization. Older CPs (50 years and above) [aOR = 0.703, CI 95% (0.598–0.812)] and those working in Bekaa and North have lower knowledge score than their counterparts. High educational level [aOR = 1.891, CI 95% (1.598–2.019)], previous experience in immunization [aOR = 3.123, CI 95% (2.652–4.161)] and working in urban areas [aOR = 3.640, CI 95% (2.544–4.717)] were positively associated with a good knowledge level.

**Conclusion:**

Most of Lebanese community pharmacists are willing to offer immunizations. The expansion of the pharmacists practice scope to include provision of immunizations required a national plan that encompasses strengthening knowledge, training, certification for eligibility to administer vaccines, enhancing pharmacovigilance and statutory reform.

## Introduction

Immunization is considered as one of the most cost-effective strategy for disease prevention [1]. Despite its known benefits, adult immunization rates worldwide fall below desired targets [2]. Many factors have been identified as barriers towards achievement of target immunization coverage rates, including general public apathy, concerns and misconceptions about the safety and efficacy of vaccines, cost, lack of access to immunizers and convenience [3]. Thus, in order to improve immunization rate, there is an urgent need to address these barriers.

Since the focus of healthcare has shifted toward prevention, pharmacists were highly encouraged to expand their practice to incorporate preventive measures including immunization [4, 5]. Besides, one of the suggested strategies to succeed in an immunization program and increase the coverage rates among adults lies in involving pharmacists in vaccine administration [6]. This extension in the practice scope can be achieved through different ways such as starting by advocating immunization through raising awareness about the benefits of vaccine among the public, particularly pharmacy visitors, discussing the patient’s immunization status and recommending vaccines during the patient’s visit to the pharmacy. Lastly, pharmacists, especially community ones, can be potential immunizers by administering vaccines in their practice setting [7].

In their attempt to increase the rate of immunization, many countries have adopted the strategy of involving non-traditional immunization providers and, thus, allowing pharmacists to administer vaccines [8]. Many studies intended to highlight the positive effects of implementing vaccinations in community pharmacies. The results of these studies showed that pharmacists increase the availability of vaccinations, accelerate immunization, effectively educate patients, affect the vaccination coverage rate, prevent new cases of diseases, and by reducing the number of diseases or complications, they bring savings to the healthcare system [9–13]. Experiences in countries, who adopted the mentioned strategy such as England, Portugal, and the United States, prove the benefits of pharmacy-driven vaccination for both patients and the healthcare system [14]. Similarly, in Canada, following the implementation of vaccination administered by pharmacists, the proportion of people vaccinated in the general population has increased significantly [15].

In Lebanon, the distribution of community pharmacies among Lebanese province is not equitable and its ownership is private on a for-profit basis [16]. According to the Lebanese Order of Pharmacists (OPL) and the Ministry of Public Health (MOPH) laws, pharmacists should be omnipresent during the pharmacy’s opening hours. However, they are not permitted by law to administer vaccines. Their role is only restricted to dispense vaccines received from pharmaceutical companies. This year has witnessed a debate about allowing pharmacists to administer influenza vaccine as applied in other countries. Subsequently, MOPH has issued a memorandum No. 149 in October 6th 2020 to assert the restricted role of CPs with regard to vaccine administration. However, based on some observations, it is commonly found that many pharmacists CPs influenza vaccines to their patients as part of their current practices. In addition, it is well known that Lebanese pharmacists are actively involved in raising awareness and public health promotion [17]. In the light of COVID-19 pandemic and the need to enroll a national vaccination plan and prior to embarking on a program expanding the scope of pharmacy practice to include the provision of immunizations, it is important to understand the intention of the Lebanese pharmacist to become an immunization provider. Such information highlights the importance of policy change and legal reform toward expanding the scope of pharmacy practice. Thus, our study aims to assess the knowledge, attitudes and beliefs of community-based Lebanese pharmacists with respect to expanding their scope of practice, in addition to their willingness to participate in vaccine administration.

## Methods

### Study design and population

A cross-sectional study using an internet-based survey was conducted during the phase preceding the arrival of the COVID-19 vaccine in Lebanon between 1 and 31st December 2020. Pharmacists were electronically invited to participate. A stratified random sampling method was used in this survey to select a representative sample of CPs from the eight Lebanese provinces. The number of CPs selected from each province was calculated using a probability-proportion to size sampling method based on the list of CPs provided by the OPL. CPs working currently in pharmacy setting and who agreed to participate to the study were eligible for participation. Exclusion criteria included: clinical pharmacists, retired CPs, those who were out of the country at the time of the survey, as well as those not practicing actually. Pharmacists who were unreachable due to change of their contact information during the time of the survey and those who refused to participate in the study were also excluded.

### Questionnaire development

A 58-item questionnaire was developed and designed specifically by the authors to assess the study objectives and to cover important aspects of pharmacists as immunizers. It was drafted, piloted and modified prior to distribution. A panel of experts involving both rural and urban pharmacists provided comments on the survey design. They were asked to provide qualitative feedback on clarity, wording, interpretability and relevance. Then, the original English draft of the questionnaire was translated and adapted to the Arabic language based on standard translation guidelines [18]. The questionnaire was pre-tested among 30 community pharmacists for survey flow, functionality, readability, comprehension of instructions, and clarity. Based upon feedback from the pre-test, minor modifications regarding readability and clarity were made to the questionnaire. Furthermore, the reliability of the questionnaire was checked. The average time for filling the survey was 12 min. The questionnaire was self-administered and consisted of close-ended questions. It was divided into 6 domains:

1. Baseline information of participants: including age, gender, profile, educational level, clinical experience and working hours in addition to information about the pharmacy (location, opening hours…).
2. Pharmacists’ willingness to administer adult vaccines.
3. Pharmacists’ knowledge about vaccination.
4. Pharmacists’ specific immunization attitudes.
5. Reasons supporting utilizing pharmacists as immunizers.
6. Elements needed to incorporate immunization in pharmacists’ practice scope.

Knowledge and attitude scores were computed. Participants’ overall knowledge and overall attitude were categorized using modified Bloom’s cut-off point as good if the score was equal or more than 60%, and poor if the score was less than 60%.

### Sample size calculation

To calculate the sample size of the study, the Raosoft sample size calculator was used. Based on a total population size of 4185 community pharmacies registered with OPL, a 95% confidence level and an absolute error of 5%, a minimal sample of 352 pharmacists was required to allow adequate power for bivariate and multivariable analyses.

### Data collection

An anonymous online questionnaire using a Google form was sent using WhatsApp or email to all community pharmacists. Then, pharmacists were contacted via phone call and notified about the survey and its purpose. The link of the study included a brief introduction to the background, the objective of the survey, and instructions for filling the questionnaire.

### Ethical considerations

A written informed consent was obtained for each participant. They were reassured that their participation is voluntary and that they are free to withdraw at any time. In addition, all information was gathered anonymously and handled confidentially. The study design assured adequate protection of study participants, and neither included clinical data about patients nor configured itself as a clinical trial. Hence, this study was exempted from ethical approval of the ministry of Public Health.

### Statistical analysis

The collected data were checked for completeness and consistency before analysis. The data were analyzed using the statistical software SPSS (Statistical Package for Social Sciences), version 22.0. A reliability analysis was done to validate each of these scores and was performed using the Cronbach’s alpha test. A coefficient of above 0.7 indicated a good internal consistency. Descriptive statistics were reported using frequency and percentages for categorical variables. Finally, the analyzed data were organized and presented in the tabular, graphical and narrative form as necessary.

## Results

### Baseline characteristics of the study participants

Table 1 shows the baseline characteristics of the participants. A total of 412 community pharmacists participated in this survey of which 54.9% are females. Most of them (62.4%) are aged less than 40 years. More than half of them (55.1%) are pharmacy owners and 55.6% have a bachelor of science (BS) in pharmaceutical sciences. With respect to their work, 43.7% of them have a work experience of more than 10 years as community pharmacist and only 23.3% have no previous experience in immunization. Regarding pharmacy distribution, the majority of pharmacists (68%) work in urban area particularly in Mount-Lebanon province (37.6%). Lastly, the majority of pharmacies where our participants work (55.30%) are opened 80–120 h per week.

**Table 1:** Baseline characteristics of the participants (*N* = 412)

	*n*	%
Gender		
Male	186	45.10%
Female	226	54.90%
Age (years)		
20–29	128	31.10%
30–39	129	31.30%
40–49	81	19.70%
Equal or more than 50	74	18.00%
Profile		
Manager	44	10.70%
Owner	227	55.10%
Staff pharmacist	141	34.20%
Educational level		
BS in pharmaceutical sciences	229	55.60%
Pharm D	102	24.80%
Master, PhD or more	81	19.70%
Years of experience		
Less than 5 years	137	33.30%
5–10 years	95	23%
More than 10 years	180	43.70%
Previous experience in immunization		
No	96	23.30%
Yes	316	76.70%
Pharmacist’s working hours per week		
24 h or less	93	23.60%
25–40 h	87	21.10%
More than 40 h	232	56.30%
Geographic location of the pharmacy		
Rural	132	32%
Urban	280	68%
Province		
Mount-Lebanon	155	37.6%
Beirut	73	17.72%
Nabatyeh + South	94	22.8%
Great Bekaa (Bekaa + Baalbeck-Hermel)	42	10.2%
Great North(North + Akkar)	48	11.65%
Number of hours/week pharmacy is open		
Less than 80 h	153	37.20%
80–120 h	228	55.30%
7 days 24/24	31	7.50%

*N* frequency, *%* percentage, *95% CI* 95% confidence interval

### Willingness to be an immunizer

**Fig. 1:**
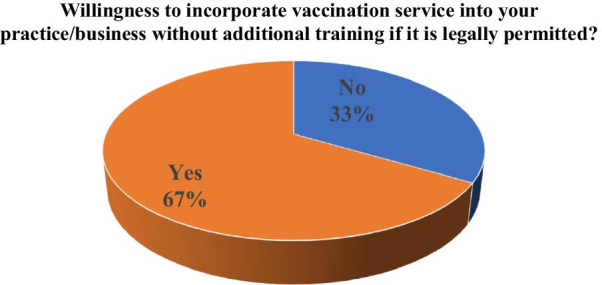
At this time, if you were legally permitted by the legislation to administer vaccines to adults, are you willing to incorporate this service into your practice/business without additional training?

**Fig. 2:**
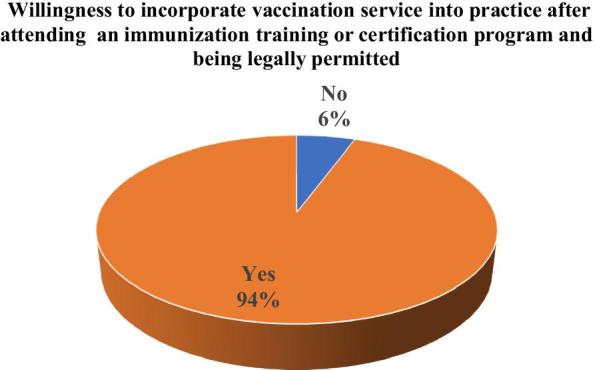
If an immunization training or certification program was available to you, and you were legally permitted by the legislation to administer vaccines to adults, are you willing to incorporate this service into your practice/business?

Of the total, 66.5% of the surveyed community pharmacists are willing to administer vaccines at this time, if they were legally permitted by the legislation to administer vaccines to adults without additional trainings. The willingness to incorporate this service into their practice rises to 94.4% if the legislation was combined to an immunization program or certification program (Figs. 1, 2).

### Pharmacists self-reported knowledge

The majority of surveyed pharmacists (89.8%) had an overall good level of knowledge (≥ 60%) and only 10.2% of them had a poor level of knowledge (< 60%). Table 2 illustrates the knowledge domains about vaccination. The majority of respondents were knowledgeable in different domains except the domain related to vaccine contraindications and precautions. The highest knowledge scales were shown in the general knowledge about vaccination (99.3%) and the domain specific to influenza vaccination (90.5%). Besides, around three-quarters of respondents were well informed about the storage, administration and adverse reactions of vaccines.

**Table 2:** Pharmacist’s knowledge domains

		Poor	Good
		*n* (%)	*n* (%)
D1	Domain 1: General knowledge	3 (0.7%)	409 (99.3%)
D2	Domain 2: Influenza vaccines	39 (9.5%)	373 (90.5%)
D3	Domain 3: Contraindications and precautions to vaccination	284 (68.9%)	128 (31.1%)
D4	Domain 4: Storage and administration of vaccine	114 (27.7%)	298 (72.3%)
D5	Domain 5: Adverse reactions following vaccination	102 (24.8%)	310 (75.2%)
	Overall knowledge	42 (10.2%)	370 (89.8%)

Table 3 describes pharmacists’ answers to vaccination knowledge items. Despite the good level of knowledge recorded in the domain related to the storage and administration of vaccines, only 45.4% of pharmacists were aware that inactivated vaccines may be administered at the same time or at any time before or after a live vaccine. Similarly, 45.4% of them were not knowledgeable about the fact that local adverse reaction such as pain, swelling, and redness at the injection site generally occurred within few hours of the injection, and are usually mild and self-limited.

**Table 3:** Pharmacists’ answers to knowledge items

#		Correct	Incorrect	I don’t Know
		*n* (%)	*n* (%)	*n* (%)
D1: General knowledge				
K1	Vaccines are critical to the prevention and control of infectious diseases outbreaks	409 (99.3%)	3 (0.7%)	0 (0%)
K2	The ingredients of the vaccine include: the antigen, adjuvants, preservatives, and stabilizers	375 (91%)	6 (1.5%)	31 (7.5%)
K3	Vaccines are safe and serious problems from the vaccine are very rare	383 (93%)	20 (7%)	9 (2.2%)
K4	Every vaccine must go through extensive and rigorous testing before it can be introduced	402 (97.6%)	6 (1.4%)	4 (1%)
K5	During the COVID-19 pandemic, vaccination continues to be critically important	396 (96.1%)	6 (1.4%)	10 (2.5%)
K6	Vaccines reduce the risks of getting a disease by working with the body’s natural defenses to build protection	412 (100%)	0 (0%)	0 (0%)
K7	Not all vaccinations may be needed in Lebanon, some may only be given prior to travel, or to people in high-risk	334 (81.1%)	78 (18.9%)	4 (1%)
K8	Following the introduction of a vaccine, close monitoring continues to detect any unexpected adverse side effects and assess the effectiveness	377 (91.5%)	22 (5.3%)	13 (3.2%)
K9	Vaccines protect us throughout life and at different ages, from birth to childhood, as teenagers, and into old age	378 (91.7%)	28 (6.8%)	6 (1.5%)
D2: Influenza vaccination				
K10	Unvaccinated people with mild symptoms of influenza can spread the disease to others	383 (93%)	22 (5.3%)	7 (1.7%)
K11	The seasonal flu vaccine protects against the most common influenza viruses including H1N1	358 (86.9%)	38 (9.2%)	16 (3.9%)
K12	Annual influenza immunization is recommended for all health-care professionals in contact with individuals in high-risk groups	404 (98.1%)	8 (1.9%)	0 (0%)
K13	Anyone can get very sick from influenza, including people who are healthy	334 (81.1%)	66 (16%)	12 (2.9%)
D3: Contraindications and precautions to vaccination				
K14	Pneumococcal vaccination is contraindicated for asplenic (without a spleen) patients	107 (26%)	103 (25%)	202 (49%)
K15	Breastfeeding is a contraindication to vaccination	292 (70.9%)	47 (11.3%)	73 (17.8%)
K16	Pregnant women who are expected to deliver during the influenza season should not receive the influenza vaccine	300 (72.8%)	56 (13.6%)	56 (13.6%)
K17	Anaphylactic reaction to a previous dose of vaccine or a vaccine component is a contraindication to further doses of the same vaccine or to the same component in other vaccines	387 (93.9%)	7 (1.5%)	18 (4.4%)
K18	Persons receiving immunosuppressive medications should not receive the influenza vaccine	204 (49.5%)	134 (32.5%)	74 (18%)
K19	Live-virus vaccines (MMRII…) should be postponed until after chemotherapy or high-dose steroid has ended	342 (83%)	25 (6.3%)	45 (10.7%)
D4: Storage and administration of vaccine				
K20	Improper storage of vaccines may affect the immune response of the vaccine recipient	384 (93.2%)	7 (1.7%)	21 (5.1%)
K21	Stabilizers protect the vaccine during storage and transportation	356 (86.4%)	26 (6.3%)	30 (7.3%)
K22	Inactivated vaccines may be administrated at the same time or at any time before or after a live vaccine	187 (45.4%)	112 (27.2%)	113 (27.4%)
K23	A person who received a live vaccine should wait 28 days before receiving another live vaccine	291 (70.6%)	48 (11.7%)	73 (17.7%)
D5: Adverse reactions following vaccination				
K24	Local adverse reaction such as pain, swelling, and redness at the injection site generally occurred within a few hours of the injection and are usually mild and self-limited	187 (45.4%)	219 (53.1%)	6 (1.5%)
K25	Systemic adverse reactions may occur following receipt of live, attenuated vaccines which must replicate to produce immunity	283 (68.7%)	85 (20.6%)	44 (10.7%)
K26	A systemic reaction is usually mild and occurs 3–21 days after the vaccine was administrated (incubation period of the vaccine)	318 (77.2%)	23 (5.5%)	71 (17.2%)
K27	Severe allergic reactions may be life-threatening but fortunately, they are rare	386 (93.7%)	10 (2.4%)	16 (3.9%)
K28	The risk of an allergic reaction can be decreased by effective screening prior to vaccination	316 (76.7%)	43 (10.5%)	53 (12.8%)
K29	Providers should report any clinically significant adverse event occurring after administration of the vaccine even if they are unsure whether the vaccine caused the event	400 (97.1%)	3 (0.7%)	9 (2.2%)

In respect of the contraindications and precautions domains of vaccine, only 26% of participants considered that pneumococcal vaccination is not contraindicated for asplenic patients.

### Pharmacists’ specific immunization attitudes

Out of all, 92.7% of surveyed pharmacists showed a positive overall attitude score toward immunization. Around 90% of them considered that vaccines produce more health benefits than health risks, and that increasing the proportion of adults who receive recommended immunizations is important. However, only 20.4% of them considered that natural infection or a healthy lifestyle are effective alternatives to vaccines.

### Reasons supporting utilizing pharmacists as immunizers

**Fig. 3:**
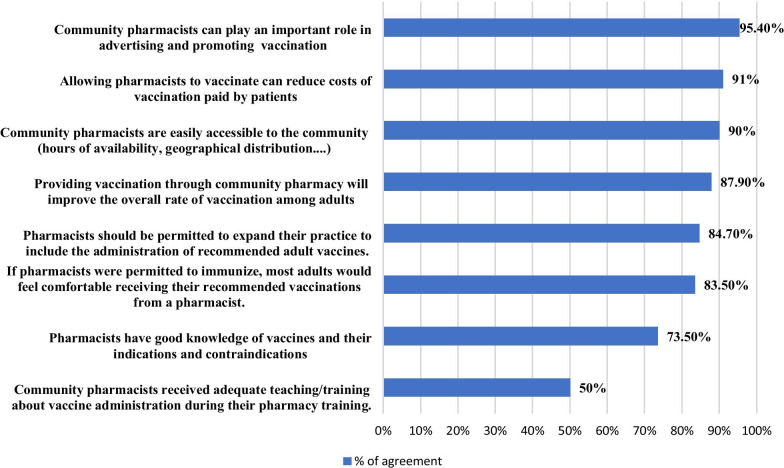
Reasons supporting utilizing pharmacists as immunizers as perceived by CPs

The majority of respondents (95.4%) agreed that community pharmacists can play an important role in advertising and promoting vaccination. Moreover, more than 90% of them considered that allowing pharmacists to vaccinate can reduce costs paid by patients, and that pharmacies are easily accessible to the community which will improve the overall vaccination rate among adults as they feel more comfortable. In addition, 84.7% of them agreed that pharmacists should be legally permitted to administer vaccines. However, only 50% of respondents agreed that pharmacists have received adequate teaching/training about vaccine administration during their education (Fig. 3).

### Elements needed for implementing immunization services in pharmacies

The main needed elements for implementing immunization services in pharmacies listed by participants were: support of health authorities (Ministry of Public Health and Order of Lebanese Pharmacists) (99.3%), statutory allowance (82.8%), patient demand (95.4%), pharmacist’s interest (96.1%) and continuous education and training workshops on immunization, safe administration and handling of vaccines (93.7%). Only 55.6% of participated pharmacists highlighted the need of formal certification in vaccine administration as requisite for allowing pharmacists to be immunizers (Table 4).

**Table 4:** Elements needed for implementing immunization services in pharmacies

		No	Yes
		*n* (%)	*n* (%)
E1	More university education and training on immunization administration for pharmacists are needed	67 (16.3%)	345 (83.7%)
E2	Formal certification in vaccine administration should be required for pharmacists	183 (44.4%)	229 (55.6%)
E3	Continuous education and training workshops on immunization, safely administration and handling of vaccines	26 (6.3%)	386 (93.7%)
E4	Financial reimbursement or adequate remuneration	66 (16%)	346 (84%)
E5	Patients demand	19 (4.6%)	393 (95.4%)
E6	Collaboration with medical clinics	86 (20.9%)	326 (79.1%)
E7	Support from medical and nursing associations	76 (18.4%)	336 (81.6%)
E8	Support of health authorities (Ministry of Public Health and Order of Lebanese Pharmacists)	3 (0.7%)	409 (99.3%)
E9	Pharmacist interest	16 (3.9%)	396 (96.1%)
E10	Legal issue	71 (17.2%)	341 (82.8%)

### Factors associated with good knowledge among CPs

Table 5 displays the factors associated with good knowledge score among CPs. Older CPs aged 50 years and above [aOR = 0.703, 95% CI (0.598–0.812)] were less knowledgeable than younger CPs aged between 20 and 39 years old. CPs with higher education level than BS in pharmaceutical sciences [aOR = 1.891, CI 95% (1.598–2.019)] and previous experience in immunization [aOR = 3.123, CI 95% (2.652–4.161)] were more likely to have a good knowledge score than those who have a BS degree in pharmaceutical sciences and who lack from previous experience in immunization. CPs working in pharmacies located in urban areas [aOR = 3.640, CI 95% (2.544–4.717)] showed better level of knowledge than their counterparts working in rural areas. Our findings showed also that CPs working in Great Bekaa [aOR = 0.703, CI 95% (0.598–0.812)] and great North have lower odds of good knowledge than those working in Beirut.

**Table 5:** Multivariable logistic regression of the factors associated with knowledge of CPs

	Overall knowledge		*P*-value	aOR	CI 95%	
	Poor	Good				
	*n* (%)	*n* (%)			Lower	Upper
Gender			0.089			
Male	16 (38.1%)	170 (61.9%)				
Female	26 (11.5%)	200 (88.5%)				
Age (years)			0.023			
20–39	15 (5.8%)	242 (94.2%)	Ref			
40–49	6 (7.5%)	75 (92.5%)	0.143	0.813	0.765	1.354
50–59	9 (16.9%)	44 (83.1%)	0.038	0.703	0.598	0.812
Equal or more than 60	12 (57.1%)	9 (42.9%)	0.021	0.604	0.478	0.756
Profile			0.207			
Manager	10 (22.7%)	34 (77.3%)				
Staff pharmacist	20 (14.2%)	121 (85.8%)				
Owner	12 (5.6%)	215 (94.4%)				
Educational level			0.012			
BS in pharmaceutical sciences	29(12.7%)	200 (87.3%)	Ref			
More than BS	13(7.1%)	170 (92.9%)	0.039	1.891	1.598	2.019
Years of experience			0.106			
< 5 years	20 (14.6%)	117 (85.4%)				
5–10 years	13(13.7%)	82 (86.3%)				
More than 10 years	9(5%)	171 (95%)				
Previous experience in immunization			0.008			
No	29 (30.2%)	67 (69.8%)	Ref			
Yes	13 (4.1%)	303 (95.9%)	< 0.001	3.123	2.652	4.161
Geographic location of the pharmacy			< 0.001			
Rural	32 (24.2%)	100 (75.6%)	Ref			
Urban	10 (3.6%)	270 (96.4%)	< 0.001	3.64	2.544	4.717
Province (pharmacy)			0.045			
Mount-Lebanon	7 (4.5%)	148 (95.5%)	Ref			
Beirut	4 (5.5%)	69 (94.5%)	0.165	0.815	0.712	1.103
Nabatyeh + South	6 (6.4%)	88 (93.6%)	0.112	0.693	0.567	1.012
Great Bekaa(Baalbeck-Hermel + Bekaa)	11 (26.2%)	31 (73.8%)	0.041	0.185	0.112	0.223
Great North(Akkar + North)	14 (29.2%)	34 (70.8%)	0.029	0.198	0.143	0.318
Number of hours/week pharmacy is open			0.131			
Less than 50 h	5 (27.8%)	13 (72.2%)				
50–120 h	29 (8%)	340 (92%)				
7 days 24/24	8 (25.8%)	23 (74.2%)				
Pharmacist’s working hours per week			0.148			
Less than 40 h	10 (10.7%)	83 (89.3%)				
More than 40 h	32 (10%)	287 (90%)				

*N* frequency, *%* percentage, *aOR* adjusted odds ratio, *95% CI* 95% confidence interval

## Discussion

This study was conducted during the preparedness phase for the roll-out of COVID-19 vaccines in Lebanon. It has particular importance during the COVID-19 pandemic as pharmacists could have a responsibility to take a prominent role in combating infectious diseases and control programs in health-care systems, in addition to their significant impact on the vaccination coverage rate. To the best of our knowledge, this is the first representative national Lebanese study aiming to explore readiness and willingness of community pharmacists to expand their practice scope into administering vaccines for adults.

The main findings in our study were that more than half of the surveyed community pharmacists are willing to start in the meantime the administration of vaccines in case they were legally permitted to do it without additional training. However, this willingness to incorporate this service into their practice rises to more than 90% if the legislation was combined to an education or certification program. This could be explained by the fact that despite their willingness to proceed, many pharmacists felt not well prepared, given that their education was not sufficient to begin at this time incorporating immunization services at their practice, and that formal certification should be required to do so. In this context, many pharmacist regulatory bodies have recognized the stipulation of proper immunization training prior to service provision, hence, the need of developing immunization training programs to ensure safe and effective administration of vaccines by pharmacists [19]. Such programs show their success in the Maritimes, in which 97% of pharmacists felt prepared to administer immunizations following completion [2].

In regard to community pharmacists’ knowledge, our results showed that the majority of respondents were knowledgeable in different domains except the domain related to vaccine contraindications and precautions. Good knowledge is crucial to expand the scope of practice of pharmacists as it supports them in providing adequate education to the public and, consequently, improving their performance and confidence in administrating vaccines. However, this study indicates that respondents lack the necessary knowledge of precautions and contraindications of vaccines, and those related to its administration such as the possibility of administration of inactivated vaccines at the same time or at any time before or after a live vaccine. Also, knowledge regarding the fact that local adverse reaction occurring at the injection site within few hours of administration and that are usually mild and self-limited, was not well recognized. These gaps in knowledge may need to be narrowed and underline a crucial need for strategies to educate community pharmacists about particular aspects of the contraindications and precautions of vaccines through continuing medical education, supplementary professional information, and additional patient educational materials [20]. It should be noted that a good proportion of the participants recognized the need for further education in this field.

It is notable that our results proved positive attitude toward utilization of pharmacists as immunizers. Most of pharmacists appraised the importance of their role in advertising and promoting vaccination among public. This is consistent with the findings of a study conducted in Italy that investigated KAP regarding vaccinations by community pharmacists in Italy [21]. Moreover, pharmacists believed that allowing them to vaccinate can reduce costs paid by patients and that pharmacies are easily accessible to the community which will improve the overall vaccination rate among adults as they feel more comfortable. Our results aligned with the findings of many studies that showed that vaccination costs were less when this service was provided by pharmacists, compared to those that were physician-administered [22]. Another study showed that immunization rates against influenza were higher for individuals aged more than years old in areas where pharmacists provided vaccinations, most likely due to improved accessibility and convenience [23, 24]. Finally, increased vaccination rate will be translated by a decrease in mortality and hospitalizations rates in elderly patients; hence, reduction of the cost related to direct medical care [25, 26].

In addition, 84.7% of pharmacists agreed that they should be legally permitted to administer vaccines. This highlights the importance to focus on statutory reform to enable pharmacists to provide vaccination under the umbrella of law [27].

Since only half of respondents agreed that pharmacists have received adequate teaching/training about vaccine administration during their education, additional training for proper immunization practices is highly recommended. This should be synchronized with regulation changes anticipated by the profession and pharmacy schools to expand the scope of practice and enable pharmacist-administered vaccination.

Regarding the factors necessary for the implementation of vaccination services in community pharmacies, participants indicated that support of health authorities (Ministry of Public Health and Order of Lebanese Pharmacists), statutory allowance, patient demand, pharmacist interest and continuous education and training workshops on immunization, safe administration and handling of vaccines are needed. Consistently, many studies found similar results with respect to essential elements identified, such as legal liability and formal education [28, 29]. With respect to patient demand, CDC estimates that almost 20% of the 2010–2011 influenza adult vaccinations were administered by pharmacists [30].

In terms of factors associated with CPs’ good knowledge, our findings showed that CPs with older age, and working in pharmacies in Bekaa and North have lower knowledge score than their counterparts. However, CPs with high educational level, previous experience in immunization and working in urban areas showed higher tendency to have good knowledge level. Our results were comparable to the findings of a study conducted in Italy that revealed that higher level of knowledge was reported among younger CPs and those with a lower number of years since degree [21]. The role of higher education in predicting better vaccination knowledge might be due that they have more opportunities to learn and receive adequate education and training through their university pharmacy curricula. Given that CPs can be a key point of contact for patients especially who live in rural areas, our findings highlighted the importance of improving the knowledge of the CPs who worked in such areas.

Continuous education and training workshops on immunization, safe administration and handling of vaccines are recommended in addition to the formal certification in vaccine administration as requisite for allowing pharmacists to be immunizers. Vaccine adverse event reporting systems are essential to ensure vaccine safety and surveillance, and maintain people's confidence in vaccines. There is need to establish immunization records which allow pharmacists to identify missed vaccinations, offer reminders to patients, and update immunization records to improve reporting of immunization coverage. Further integration of such systems and procedures across healthcare providers are needed for pharmacists to be incorporated in the overall health system to present a united front in offering vaccination services. Lastly, it is necessary to prepare standards as well as a legal framework for the provision of such services.

### Limitations of the study

Some limitations of this study should be acknowledged. First, the cross-sectional design of this study does not allow us to infer causality. Second, our study relies on community pharmacists’ self-reported information, which makes it prone to the disadvantages of desirability biases. Furthermore, this online questionnaire might have favored a selection bias since it might only allow the participation of community pharmacists who have access to online resources. Lastly, recall bias could have occurred particularly for the questions related to past behavior.

## Conclusion

Most of Lebanese community pharmacists are willing to offer immunizations. Thus, the expansion of CPs scope of practice to include the provision of vaccine required the implementation of a national plan. This plan can encompass the following pillars that should be incorporated in parallel: strengthening knowledge and education about the topic for graduated pharmacist, incorporating vaccines and immunization in the academic curricula, offering post-graduate certificate and diploma for eligibility to administer vaccines with periodic re-certification, enhancing the role of pharmacovigilance and reporting within this scope and, statutory reform. Future studies are needed to examine pharmacists’ perceived barriers about immunization, as well as the determinants of their willingness to administer vaccines.

## Data Availability

Data are available from the corresponding authors upon reasonable request.
